# Effects of Rearfoot Eversion on Foot Plantar Pressure and Spatiotemporal Gait Parameters in Adolescent Athletes

**DOI:** 10.3390/healthcare11131842

**Published:** 2023-06-25

**Authors:** Hironori Fujishita, Yasunari Ikuta, Noriaki Maeda, Makoto Komiya, Masanori Morikawa, Satoshi Arima, Tetsuhiko Sakamitsu, Hiromune Obayashi, Kouki Fukuhara, Kai Ushio, Nobuo Adachi

**Affiliations:** 1 Department of Sports Medical Center, Hiroshima University Hospital, Hiroshima 734-8551, Japan; 2 Department of Orthopaedic Surgery, Graduate School of Biomedical and Health Sciences, Hiroshima University, Hiroshima 734-8551, Japan; 3 Department of Sports Rehabilitation, Graduate School of Biomedical and Health Sciences, Hiroshima University, Hiroshima 734-8553, Japan; 4 Department of Rehabilitation Medicine, Hiroshima University Hospital, Hiroshima 734-8551, Japan

**Keywords:** adolescent athlete, foot morphology, gait, plantar foot pressure, rearfoot eversion, spatiotemporal gait analysis

## Abstract

Background: Foot malalignment can augment the risk of lower-extremity injuries and lead to musculoskeletal disorders. This study aimed to clarify the contribution of rearfoot alignment to plantar pressure distribution and spatiotemporal parameters during gait in healthy adolescent athletes. Methods: This retrospective study included 39 adolescent athletes who were divided into the rearfoot eversion and control groups according to a leg heel angle of 7°. A total of 78 legs were analyzed (45 and 33 legs in the rearfoot eversion [women, 53.3%] and control groups [women, 48.5%], respectively). Gait was assessed using an in-shoe plantar pressure measuring system and a wearable inertial sensor. Results: The foot plantar pressure distribution in the hallux was higher in the rearfoot eversion group than that in the control group (*p* = 0.034). Spatiotemporal parameters showed that the foot pitch angle at heel strike was significantly larger in the rearfoot eversion group than that in the control group (24.5° vs. 21.7°; *p* = 0.015). Total sagittal range of motion of the ankle during the stance phase of gait was significantly larger in the rearfoot eversion group than that in the control group (102.5 ± 7.1° vs. 95.6 ± 15.8°; *p* = 0.020). Logistic regression analysis revealed that plantar pressure at the hallux and medial heel and foot pitch angle at heel strike were significantly associated with rearfoot eversion. Conclusions: Our findings suggest that rearfoot eversion affects the gait patterns of adolescent athletes. Notably, leg heel angle assessment, which is a simple and quick procedure, should be considered as an alternative screening tool for estimating plantar pressure and spatiotemporal gait parameters to prevent sports-related and overuse injuries in adolescent athletes.

## 1. Introduction

Young athletes are more vulnerable to sports-related injuries than mature adult athletes owing to physical and physiological differences [1], and the highest incidence of physical injuries caused unintentionally during sports and recreational activities has been reported in children aged 10–14 years [2]. The ankle and knee joint constitute the highest number of extremity injuries, and ankle sprains are common sports-related injuries in adolescent athletes [1,3,4]. The potentially modifiable risk factors for sports injuries in children and adolescents include flexibility, muscle strength, joint stability, and postural stability or proprioception [5]. Among high-school basketball players, those with higher postural sway scores demonstrated nearly seven times as many ankle sprains as those with good postural stability [6].

Previous reports have demonstrated a correlation between postural stability and foot morphology in young adults [7,8,9]; however, discussion of these aspects in adolescents is lacking. Foot malalignment can lead to musculoskeletal disorders [10,11], and excessive foot pronation has been proposed as an anatomical risk factor for lower-extremity injuries [12]. Therefore, the clinical screening of foot morphology is essential to prevent sports-related injuries and further pathological deformations in adolescent athletes. The development of foot structure and function with age should be considered in adolescents, as foot arch function develops with age, and the foot acquires an adult-like form at 12–14 years; however, the complete adult form is acquired at 18 years of age [13]. The rearfoot angle matures earlier than the foot arch. Normal values for the rearfoot angle in healthy adults lie between 3.5 and 7.0°, and the adult value is attained at 5–7 years of age [14]. Furthermore, a regression analysis demonstrated that the rearfoot angle was a significant predictor of the clinically defined foot type and the rearfoot angle accounted for 78% of the variance in foot type assessment [15]. Therefore, in this study, the extent of foot malalignment was assessed by determining the leg heel angle (LHA), which is used clinically to evaluate rearfoot eversion (RE). Poor postural stability has been reported during single-leg standing on a dynamic platform in adolescent athletes with RE [16]; however, their findings clarified the association between RE and static postural control alone. It is essential to investigate how RE affects gait function, such as acting loads, range of motion, and cycle [17], as this may aid in the prevention of sports-related and lower extremity overuse injuries in adolescent athletes.

Foot plantar pressure and spatiotemporal gait assessments are used to assess foot function and contact patterns [18]. Age-specific plantar pressure distribution in children aged 6–14 years has been reported, and older children have higher force-time integral (FTI), contact area, and peak plantar pressure values than younger children [19]. Compared to a younger age, 14 years is a critical age for the alteration of plantar pressure distribution [10]. Orthopedic foot disorders change the plantar pressure distribution, which provides important clinical information to better understand the alteration of foot function [11]. Foot-strike patterns are associated with the anatomical location of running-related lower extremity injuries in adolescent runners [20]. Despite this interest, to the best of our knowledge, no study has examined the correlation between foot alignment, plantar pressure, and gait function in adolescent athletes. Although RE affects adjacent joint kinematics and leads to forefoot pronation and the internal rotation of the tibia through the musculoskeletal kinematic chain [21], its impact on gait function in adolescent athletes is not fully understood. We hypothesized that adolescent athletes present a characteristic foot pressure distribution and gait function depending on the extent of RE. This study aimed to clarify the impact of RE on foot pressure distribution and spatiotemporal parameters during gait in healthy adolescent athletes with versus without RE.

## 2. Materials and Methods

### 2.1. Participants

This retrospective study included 46 adolescent athletes who underwent medical and physical checkups at the Sports Medical Center of Hiroshima University Hospital in August 2020. The participants were selected by the Hiroshima City Sports Association as part of a specially designed program in 2020 aimed at improving player strength and preventing sports-related injuries. Each participant regularly performed >60 min/day of physical activity throughout the week. None of the participants had a history of cardiometabolic disease or orthopedic surgery and no restriction of musculoskeletal or joint motion was observed.

**Figure 1:**
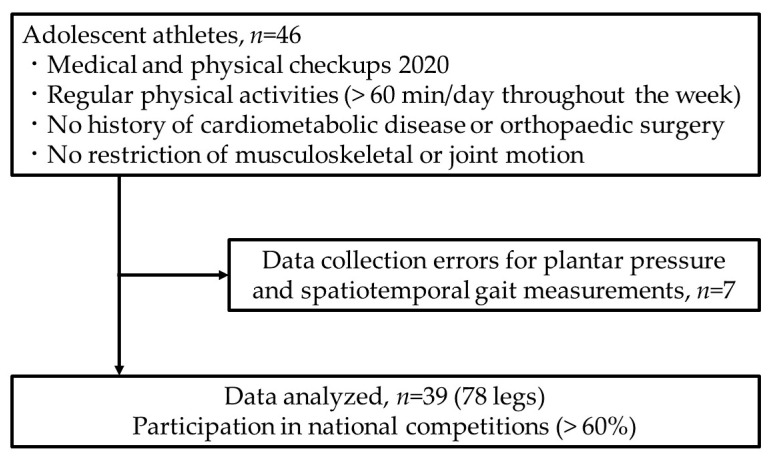
Study participants.

The exclusion criteria were as follows: musculoskeletal injury within 3 months prior to the medical checkup, which required cessation of sports activities, and data collection errors regarding plantar pressure and spatiotemporal gait measurement. Data collection errors occurred for foot plantar pressure and spatiotemporal gait measurements in seven participants. Consequently, 78 legs of 39 adolescent athletes (19 boys and 20 girls) with a mean age of 13.7 years (range: 13–15 years) were included in the study. More than 60% of the athletes participated in national competitions, including basketball, handball, hockey, kendo, rugby, and table tennis (Figure 1).

### 2.2. Measures

#### 2.2.1. Body Composition Measurement

Our research group has previously reported a protocol for body-composition measurements [22]. All participants skipped breakfast, fasted for at least 9 h, and refrained from drinking water for 30 min before the measurements. Body height and mass were measured using a Seca 213 portable stadiometer (Seca NIHON, Chiba, Japan) and a FitScan FS-101 (TANITA, Tokyo, Japan), respectively. Body composition was measured in the supine position using bioelectrical impedance analysis with an InBody S10 (InBody Co., Seoul, Korea). After pretreating the skin, the electrodes were placed on the first and third fingers of each hand and both ankles.

#### 2.2.2. Foot Morphological Measurement

Rearfoot alignment was assessed using the LHA formed by the bisection of the distal one-third of the leg and a longitudinal line that bisected the posterior aspect of the calcaneus [23]. Participants stood barefoot on a 30 cm tall box in a completely weight-bearing double-limb standing position. A physical therapist measured the angle by using a two-arm goniometer. The participants were divided into two groups according to their rearfoot angles, RE and control groups, with rearfoot angles ≥7° and <7°, respectively, according to a previous report [14].

To assess the navicular index and navicular drop, vertical changes in the navicular tuberosity were measured using a three-dimensional laser foot scanner (FSN 2100, Dream GP Inc., Osaka, Japan), a reliable equipment for anthropometric foot data collection (Intraclass Correlation Coefficients: 0.94–0.99) [24]. Dedicated markers were attached to both navicular tuberosities, with the participants seated on a chair. One side of the foot was placed inside the foot scanner and the ankle and knee joints were flexed at 0° and 90°, respectively.

Ultrasonographic measurements of the intrinsic foot muscles were performed as described previously [25,26]. Images of the intrinsic foot muscle and plantar fascia morphology with thickness and cross-sectional area (CSA) were obtained using B-mode ultrasonography (HI VISION Avius; Hitachi Aloka Medical, Tokyo, Japan) with an 8-MHz linear array probe to visualize a continuous sagittal ultrasound image. The selected thickness and CSA of the muscle’s thickest part were measured using B-mode ultrasonography following the same procedure used in previous studies [27,28]. The following were measured in the prone position with the participants’ ankles in a neutral position and their knees flexed at 90°: (1) thickness of the abductor hallucis (AbH), flexor hallucis brevis (FHB), flexor digitorum brevis (FDB), and plantar fascia and (2) CSA of the AbH, FHB, and FDB.

#### 2.2.3. Muscle Strength and Joint Mobility Measurement

Muscle strength was evaluated, including isokinetic knee extension/flexion, isokinetic ankle dorsiflexion, plantar flexion, and isometric toe flexion. Biodex System 4 (Biodex Medical Systems Inc., Shirley, NY, USA) was used to assess isokinetic muscle strength. Muscle strength was measured using the protocol described in our previous report [29]. Participants performed maximum knee extension and flexion at an angular velocity of 60°/s. For ankle dorsiflexion and plantarflexion measurement, the participant was seated, and the test leg was elevated with the support arm. Each foot was positioned and fixed to a footboard. Participants performed maximum ankle dorsiflexion and plantarflexion at an angular velocity of 60°/s. The maximum peak torque was recorded. Each value, which was calibrated with body mass, was used to correct for body mass during data analysis.

Toe-flexor strength was assessed in the sitting position by using a toe grip dynamometer (T.K.K.3361; Takei Scientific Instruments, Niigata, Japan) consisting of strain-gauge force transducers. The methods are described in detail in a previous study [25]. Measurement of toe flexor strength was performed with maximum force held for 5 s, and the average of three measurements was used for analysis.

The passive range of motion of the ankle was measured with the knee extended in the supine position. One physical therapist held the maximum dorsiflexion and plantar flexion positions, while the other measured the angle using a goniometer.

#### 2.2.4. Gait Functional Assessment

The gait was assessed in two ways using plantar pressure measurements and accelerometric systems. Plantar pressure and spatiotemporal data were measured simultaneously during one gait trial. However, the pressure and spatiotemporal sensors were not synchronized owing to technical limitations of both systems. All participants were instructed to walk at a comfortable speed on a 30 m line from a standing position. The participants wore their own sports shoes to aid reproduction of their usual gait patterns during the gait function assessment. The methodology for each system is outlined as follows.

#### 2.2.5. Plantar Pressure Measurement

Plantar pressure distribution was evaluated using the Pedar-X system (Novel, Munich, Germany). This in-shoe plantar pressure system comprised 99 capacitive sensors sampled at 100 Hz and was attached to the participants’ waist using a belt. The left and right cables were placed on each ankle using elastic Velcro. This plantar pressure system has been shown to be valid and reliable [30,31]. Data from 16 steps (from the middle of the 30 m walking test, excluding at least two steps from the beginning and end) per participant were analyzed using Pedar software according to the manufacturer’s instructions.

**Figure 2:**
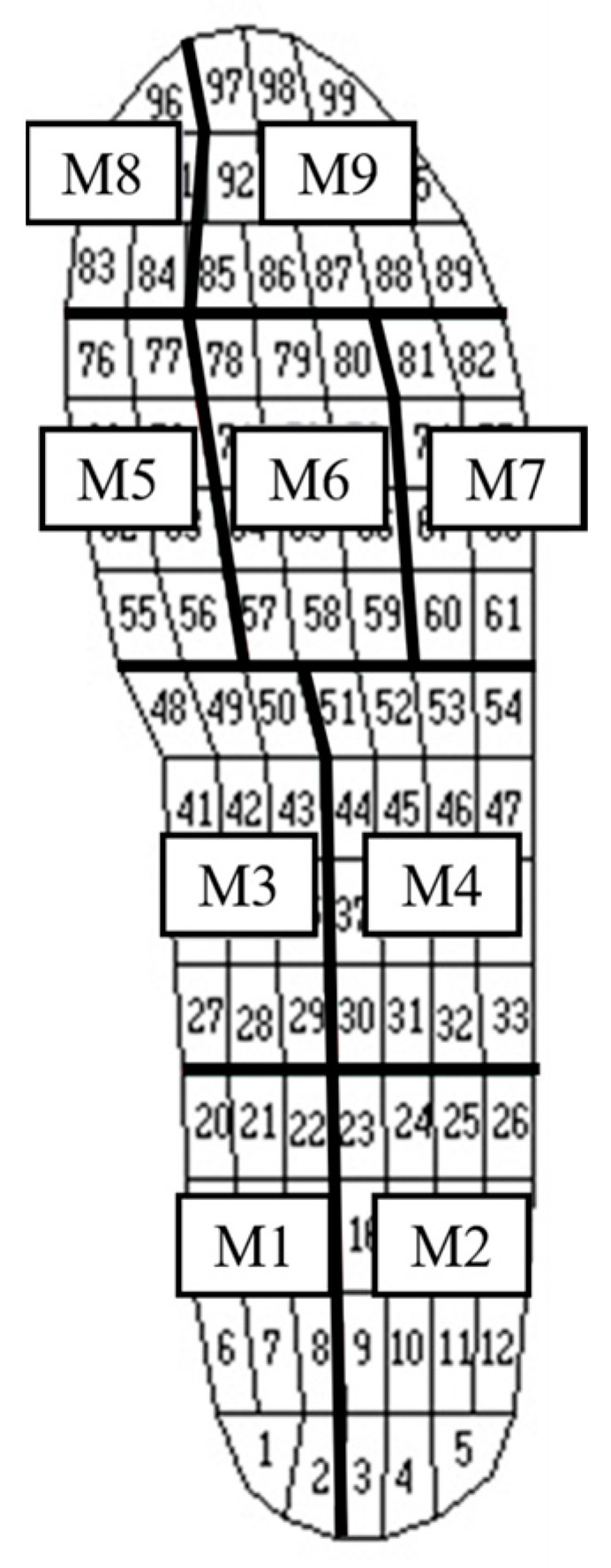
The nine areas of foot plantar pressure. The sole of the foot divided into nine area according to 99 sensors.

Peak plantar pressure was also analyzed in the following nine areas: medial heel (M1), lateral heel (M2), medial center (M3), lateral center (M4), medial metatarsal (M5), center metatarsal (M6), lateral metatarsal (M7), hallux (M8), and second to fifth toes (M9) (Figure 2). For each of the nine areas, the peak plantar pressure corresponded to the sum of the maximum pressure values generated during gait by all 99 sensors. The contact area and FTI were calculated using Pedar Systems.

#### 2.2.6. Spatiotemporal Measurement

Two inertial sensor systems (Physilog5, Gait Up Ltd., Lausanne, Switzerland) were placed on the anterior aspect of each shoe and fixed with shoelaces. The spatiotemporal parameters of both feet were collected at 128 Hz sampling rates. Both sensors were wirelessly synchronized, and each comprised a three-dimensional accelerometer (range ±4 g) and gyroscope (range ±1000°/s). The validity and reliability of this device have been well demonstrated, including its use in adolescents [32,33,34]. Data analysis was performed by excluding two gait cycles from the beginning and end of the 30 m walking. The temporal parameters used were the gait cycle time (GCT), cadence, stance, and swing ratios. The stance phase was subdivided into three ratios: load ratio (LDr), foot flat ratio (FFr), and push ratio (Pur). The load phase is the duration between the heel and toe strikes. The foot flat phase indicates the action of grounding the foot flat. The push phase is the duration between the heel-off and toe-off. These ratios were generated using the PhysiGait Lab system. The spatial parameters included stride length, speed, foot pitch angle at heel strike (HSP), foot pitch angle at toe-off (TOP), and peak angular velocity during the swing (peak-swing). The foot pitch angle is calculated as the angle between the ground and foot at heel strike and toe-off on a vertical plane. The peak angular velocity is the maximum angular velocity during the swing phase between the maximum heel clearance and the minimum toe clearance.

### 2.3. Statistical Analysis

The data were analyzed using SPSS 25 (IBM Japan Co., Ltd., Tokyo, Japan). The Shapiro–Wilk test was used to test the normality of the data distribution. Normally and non-normally distributed variables are presented as mean ± standard deviation. According to the normality of each measurement, Student’s *t*-test or Welch’s *t*-test was used to test between-group differences.

Logistic regression analysis was performed to investigate the association between RE and gait parameters. RE defined by a rearfoot angle ≥7° was set as a dependent variable, and the parameters with significant between-group differences were used as explanatory variables. To avoid overfitting, a regression analysis was performed for plantar pressure and spatiotemporal parameters, as in Models 1 and 2, respectively. We repeated the logistic regression analysis to determine the final model with explanatory variables that were significant in Models 1 and 2. Statistical significance was set at *p* < 0.05.

## 3. Results

The RE group comprised 33 legs (48.5% female), and the control group consisted of 45 legs (53.3% female). The mean LHA values were 9.1 ± 1.9° and 4.1 ± 1.7° in the RE and control groups, respectively. The participant characteristics are presented in Table 1. Although there were statistically significant differences in age (*p* = 0.038), the mean values only had a difference of 0.5 years. Body composition measurements revealed no physical or compositional characteristics. The navicular index was significantly higher in the RE group than that in the control group (*p* = 0.046), although the navicular drop was not significantly different between the groups (*p* = 0.717). No significant differences were found in the intrinsic foot muscle thickness or CSA between the groups (Table 2). The mean dorsiflexion strength in the RE group was significantly higher than that in the control group (44.9 ± 16.7 Nm/kg vs. 37.6 ± 8.4 Nm/kg; *p* = 0.041). No significant differences were observed between passive ankle dorsiflexion and plantarflexion.

The total contact area and peak plantar pressure in the nine areas were not significantly different between the groups. However, some areas exhibited different reactions; the heel pressure was significantly lower in the RE group than that in the control group at M1 (*p* = 0.026) and M2 (*p* = 0.021). The metatarsal bar areas, such as M5, M6, and M7, were smaller than those in the control group. In contrast, M8 expression was significantly higher in the RE group than that in the control group (*p* = 0.034). The FTI was significantly lower in the RE group than that in the control group.

Spatiotemporal analysis revealed significant differences in GCT and HSP between the groups. Although no significant differences were observed with respect to gait speed (1.2 ± 0.1 m/s vs. 1.1 ± 0.2 m/s; *p* = 0.126), the HSP was significantly larger in the RE group than that in the control group (24.5° vs. 21.7°; *p* = 0.015). Additionally, total sagittal range of motion in ankle during stance phase of gait was significantly larger in the RE group than that in the control group (102.5 ± 7.1° vs. 95.6 ± 15.8°; *p* = 0.020).

The correlations among rearfoot angle, foot morphology, plantar pressure, and spatiotemporal evaluation are shown in Table 3. Logistic regression analysis revealed that the LHA was associated with medial heel pressure, first toe pressure, and HSP.

## 4. Discussion

This study was conducted to investigate the effects of RE on plantar pressure distribution and spatiotemporal gait function in healthy adolescent athletes. Our results revealed that excessive RE affected gait patterns, resulting in high pressure in the hallux, low pressure in the medial heel, and increased HSP.

Several factors affect plantar pressure distribution and spatiotemporal variables, such as age, sex, foot development, foot morphology, and foot function [11,18,35]. Foot pronation increases the RE during walking [12]. Adult participants with planus feet showed higher peak pressure at the hallux and lower peak pressure at the 5th metatarsophalangeal joint during gait than those with normal feet. Foot posture was classified according to the foot posture index, arch index, and truncated normalized navicular height [36]. The foot arch structure develops over time and reaches the adult form at approximately 18 years of age. The rearfoot angle matures earlier than the foot arch; thus, LHA may be more suitable for foot posture assessment in adolescents. In contrast to our study, a previous study demonstrated that valgus rearfoot alignment increased plantar pressures at the medial rearfoot and midfoot during running in young adults [37]; however, rearfoot alignment was measured in a non-weight-bearing condition, and the participants’ age and experimental task differed from those in the current study. RE is mainly caused by subtalar joint pronation, which affects both the truss and windlass mechanisms via a loosely packed joint position. Foot pronation provided gait advantages regarding enhanced shock absorption at the heel, in which the peak pressures of the medial and lateral heels were significantly lower than those in the control group. However, RE has the disadvantage of reducing the anterior displacement of the center of pressure in the loading response to the mid-stance phase [38]. Our results showed a significantly high plantar pressure in the hallux of the RE group. These findings suggest that insufficient windlass function during the latter half of the stance phase and high plantar pressure in the hallux may have been induced by the requirement to generate propulsion in the RE group. Furthermore, this gait modification can lead to a short GCT and increased HSP to maintain gait speed during the swing phase. Consequently, high dorsiflexion muscle strength might be demonstrated in adolescent athletes with excessive RE. Additionally, a previous study revealed that flatfoot has a small second ground reaction force and is powerless during gait [39]. In the present study, FTI was significantly lower in the RE group than that in the control group, which may indicate that participants with RE have kinetic disadvantages for forward propulsion.

In this study, the navicular index was significantly higher in the RE group than that in the control group. However, no significant differences were found in navicular displacement, foot intrinsic muscle CSA, and foot muscle thickness between the groups. The thicknesses of the intrinsic and extrinsic foot muscles are not related to the severity of pronated foot deformity [40]. Individuals with symptomatic pronated feet demonstrated a smaller CSA of the flexor digitorum longus and AbH and thinner peroneus muscles and AbH than their asymptomatic counterparts [41]. Our findings of foot intrinsic muscle CSA and thickness could be partially explained by the fact that the study participants were healthy and asymptomatic adolescent athletes with no limitations in their physical activities.

Our findings concerning differences in plantar pressure distribution will be beneficial for preventing sports-related and overuse injuries and foot disorders in adolescents. This study suggests that healthy adolescent athletes with excessive RE have high plantar pressure in the hallux and low peak pressure in the metatarsal and heel areas, as well as spatiotemporal gait differences. Adolescent athletes sometimes experience turf toe, which can develop into hallux valgus due to repeated high-intensity physical activities [42,43]. The peak plantar pressure is high in the hallux and low in the metatarsal area during walking in athletes with turf toe and asymptomatic younger adults with mild hallux valgus [44,45]. Attention should be given to the fact that, even in healthy adolescent athletes who participated in this study, there were gait functional differences in feet with high RE. LHA assessment should be considered as a screening tool to determine foot alignment in adolescent athletes with the potential to estimate plantar pressure distribution and spatiotemporal parameters and is essential to prevent sports-related injuries and musculoskeletal disorders at a young age. Further prospective studies are required to clarify the association among specific plantar pressure distributions, gait patterns, lower extremity injuries, and foot disorders in adolescent athletes with and without RE.

This study has several limitations. First, foot alignment was assessed only through the body surface, including the LHA, navicular index, and navicular drop tests. Imaging assessments, such as plain radiographs, were not conducted to avoid radiation exposure, and further studies are required to determine the relationship between the RE angle and flatfoot deformities. LHA assessment is an easy and noninvasive method in the clinical and sports fields and is suitable for young adults with feet in the growing stage. Hence, it was meaningful to use the LHA as a parameter. Second, the LHA was measured only in a static posture. Future studies should evaluate dynamic changes in the LHA during gait using a motion-capture system. In addition, computational modeling with finite element analysis may assist plantar pressure assessments through von Mises stress distributions on the plantar aspect of the foot in a gait cycle [46]. Third, adolescents who are not involved in a particular sport should be used as a control group to clarify the effects of sports participation on gait function. The number of participants included in this study was small; hence, we could not divide the participants according to the type of sports. Therefore, further studies focusing on specific types of athletes and control groups of non-athlete adolescents are required. Fourth, each participant was assessed while wearing shoes. Gait assessment may have been affected by the form and function of the participants’ shoes; however, the participants were allowed to wear their shoes to observe their natural gaits.

## 5. Conclusions

High hallux pressure, low medial heel pressure, and increased foot pitch angle at heel strike during gait were identified in adolescent athletes with excessive RE who had high isokinetic muscle strength during ankle dorsiflexion. LHA measurement, which is a simple and quick approach, has the potential to predict plantar pressure distribution and gait function. Therefore, these assessments should be applied to adolescent athletes to screen foot function during gait and prevent sports-related injuries.

## Figures and Tables

**Table 1:** Characteristics of participants.

	Control Group (*n* = 45)	RE Group (*n* = 33)	*p* Value
Female (%)	24 (53.3)	16 (48.5)	0.819
Age (years)	13.9 ± 0.88	13.4 ± 0.97	0.038
Body height (cm)	161.4 ± 7.22	160.7 ± 5.24	0.663
Body mass (kg)	55.5 ± 12.07	52.7 ± 8.19	0.293
BMI (kg/m^2^)	21.2 ± 3.46	20.4 ± 2.43	0.373
Body fat percent (%)	20.0 ± 7.45	17.6 ± 7.01	0.142

Abbreviation: RE, rearfoot eversion, BMI, body mass index.

**Table 2:** Results of foot morphology and gait assessment.

	Control Group (*n* = 45)	RE Group (*n* = 33)	*p* Value
Foot morphological parameters			
Navicular Index	6.9 ± 1.11	7.4 ± 1.07	0.046
Navicular drop (mm)	8.4 ± 2.88	8.2 ± 2.94	0.717
AbH CSA (mm^2^)	243.5 ± 56.62	241.5 ± 72.50	0.895
AbH thickness (mm)	11.4 ± 2.07	11.4 ± 2.20	0.846
FDB CSA (mm^2^)	191.8 ± 48.70	206.1 ± 52.94	0.222
FDB, thickness (mm)	7.5 ± 1.56	7.9 ± 1.54	0.321
FHB CSA (mm^2^)	208.7 ± 31.68	218.4 ± 32.02	0.187
FHB, thickness (mm)	10.5 ± 1.91	10.4 ± 1.42	0.842
Muscle strength parameters			
Ankle plantarflexion (Nm/kg)	80.6 ± 25.54	87.0 ± 26.53	0.291
Ankle dorsiflexion (Nm/kg)	37.6 ± 8.41	44.9 ± 16.72	0.041
Toe flexor strength (kg)	19.1 ± 5.69	20.3 ± 5.52	0.671
Ankle range of motion			
Plantarflexion (°)	47.0 ± 11.20	49.4 ± 5.97	0.542
Dorsiflexion (°)	11.1 ± 10.22	9.4 ± 5.27	0.823
Plantar pressure variables			
Total contact area (cm^2^)	141.2 ± 12.75	138.1 ± 11.18	0.266
FTI (N-s)	1973.4 ± 481.98	1667.9 ± 464.27	0.006
WPP (kPa)	279.6 ± 32.58	251.2 ± 76.07	0.517
M1 (kPa)	111.3 ± 25.71	99.2 ± 19.69	0.026
M2 (kPa)	115.9 ± 22.61	103.8 ± 22.32	0.021
M3 (kPa)	22.8 ± 14.44	24.8 ± 12.32	0.515
M4 (kPa)	64.2 ± 15.63	57.6 ± 14.63	0.062
M5 (kPa)	119.6 ± 40.39	100.8 ± 33.45	0.033
M6 (kPa)	115.8 ± 28.21	98.8 ± 27.68	0.010
M7 (kPa)	103.1 ± 24.34	86.8 ± 19.06	0.002
M8 (kPa)	131.7 ± 54.12	158.4 ± 54.37	0.034
M9 (kPa)	59.5 ± 20.57	63.6 ± 18.91	0.376
Spatiotemporal variables			
GCT (s)	1.13 ± 0.07	1.10 ± 0.05	0.034
cadence (steps/min)	107.1 ± 6.83	109.7 ± 4.76	0.050
stance (% gct)	62.4 ± 2.00	62.4 ± 1.56	0.986
swing (% gct)	37.6 ± 2.00	37.6 ± 1.56	0.986
LDr (% stance)	10.0 ± 2.34	11.1 ± 2.26	0.052
FFr (% stance)	54.5 ± 5.81	52.4 ± 5.23	0.104
Pur (% stance)	35.5 ± 5.17	36.5 ± 5.10	0.219
slength (m)	1.3 ± 0.12	1.3 ± 0.09	0.262
speed (m/s)	1.1 ± 0.16	1.2 ± 0.10	0.126
HSP (°)	21.7 ± 6.62	24.5 ± 3.12	0.015
TOP (°)	−73.8 ± 10.90	−78.0 ± 5.61	0.088
peak-swing speed (°/s)	370.5 ± 64.60	395.2 ± 30.70	0.068

Abbreviation: RE, rearfoot eversion; AbH, abductor halluces; CSA, cross-sectional area; FDB, flexor digitorum brevis; WPP, whole area of peak pressure; M1, medial heel; M2, lateral heel; M3, medial center; M4, lateral center; M5, medial metatarsal; M6, center metatarsal; M7, lateral metatarsal; M8, hallux; M9, second to fifth toes; GCT, gait cycle time; LDr, load ratio; FFr, foot flat ratio; Pur, push ratio; slength, stride length; HSP, foot pitch angle at heel strike; TOP, foot pitch angle at toe-off; peak-swing, maximum angular velocity during swing.

**Table 3:** Association of leg heel angle and gait parameters.

	Model 1			Model 2			Final Model		
	OR	95% CI	*p* Value	OR	95% CI	*p* Value	OR	95% CI	*p* Value
Plantar pressure parameters									
M1 (kPa)	0.974	0.952–0.998	0.030				0.965	0.941–0.990	0.007
M2 (kPa)	0.994	0.957–1.031	0.731						
M5 (kPa)	0.989	0.973–1.005	0.173						
M6 (kPa)	1.000	0.968–1.033	0.999						
M7 (kPa)	0.969	0.944–0.994	0.015				0.975	0.950–1.001	0.056
M8 (kPa)	1.012	1.002–1.022	0.019				1.011	1.001–1.021	0.033
Spatiotemporal parameters									
GCT (s)				0.034	0.000–329.306	0.470			
HSP (°)				1.107	1.008–1.216	0.033	1.134	1.003–1.283	0.015
TOP (°)				0.953	0.881–1.031	0.229			
peak-swing (°/s)				0.987	0.961–1.014	0.341			

CI, confidence interval; GCT, gait cycle time; HSP, foot pitch angle at heel strike; M1, medial heel; M2, lateral heel; M5, medial metatarsal; M6, center metatarsal; M7, lateral metatarsal; M8, hallux; OR, odds ratio; peak-swing, maximum angular velocity during swing; TOP, foot pitch angle at toe-off.

## Data Availability

The data presented in this study are available on request from the corresponding author.
